# An efficient method for cell sheet bioengineering from rBMSCs on thermo-responsive PCL-PEG-PCL copolymer

**DOI:** 10.1186/s13036-023-00346-8

**Published:** 2023-04-06

**Authors:** Sevil Vaghefi Moghaddam, Fatemeh Abedi, Hajie Lotfi, Roya Salehi, Abolfazl Barzegar, Mohamadreza Baghaban Eslaminejad, Mostafa Khalili, Effat Alizadeh

**Affiliations:** 1 Clinical Research Development, Unit of Tabriz Valiasr Hospital, Tabriz University of Medical Sciences, Tabriz, Iran; 2 Cellular and Molecular Research Center, Research Institute for Prevention of Non-Communicable Diseases, Qazvin University of Medical Sciences, Qazvin, Iran; 3 Department of Medical Nanotechnology, Faculty of Advanced Medical Sciences, Tabriz University of Medical Sciences, Tabriz, Iran; 4 Department of Animal Biology, Faculty of Natural Sciences, University of Tabriz, Tabriz, Iran; 5 Department of Stem Cells and Developmental Biology, Cell Science Research Center, Royan Institute for Stem Cell Biology and Technology, ACECR, Tehran, Iran; 6 Department of Medical Biotechnology, Faculty of Advanced Medical Sciences, Tabriz University of Medical Sciences, Tabriz, Iran

**Keywords:** Cell sheets, PCL-PEG-PCL, Sol-Gel, Sodium Selenite, Stem cell

## Abstract

Utilizing both medium enrichment and a thermos-responsive substrate to maintain the cell-to-cell junctions and extracellular matrix (ECM) intact, cell sheet technology has emerged as a ground-breaking approach. Investigating the possibility of using sodium selenite (as medium supplementation) and PCL-PEG-PCL (as vessel coating substrate) in the formation of the sheets from rat bone marrow-derived mesenchymal stem cells (rBMSCs) was the main goal of the present study. To this end, first, Polycaprolactone-co-Poly (ethylene glycol)-co-Polycaprolactone triblock copolymer (PCEC) was prepared by ring-opening copolymerization method and characterized by FTIR, ^1^ H NMR, and GPC. The sol-gel-sol phase transition temperature of the PCEC aqueous solutions with various concentrations was either measured. Next, rBMSCs were cultured on the PCEC, and let be expanded in five different media containing vitamin C (50 µg/ml), sodium selenite (0.1 µM), vitamin C and sodium selenite (50 µg/ml + 0.1 µM), Trolox, and routine medium. The proliferation of the cells exposed to each material was evaluated. Produced cell sheets were harvested from the polymer surface by temperature reduction and phenotypically analyzed via an inverted microscope, hematoxylin and eosin (H&E) staining, and field emission scanning electron microscopy (FESEM). Through the molecular level, the expression of the stemness-related genes (Sox2, Oct-4, Nanog), selenium-dependent enzymes (TRX, GPX-1), and aging regulator gene (Sirt1) were measured by q RT-PCR. Senescence in cell sheets was checked by beta-galactosidase assay. The results declared the improved ability of the rBMSCs for osteogenesis and adipogenesis in the presence of antioxidants vitamin C, sodium selenite, and Trolox in growth media. The data indicated that in the presence of vitamin C and sodium selenite, the quality of the cell sheet was risen by reducing the number of senescent cells and high transcription of the stemness genes. Monolayers produced by sodium selenite was in higher-quality than the ones produced by vitamin C.

## Introduction

Over the last few years, medical biotechnology has gained substantial technological progress in. In this regard, both cell culture technology and chemical thermosensitivity technology would cause to produce the cell sheet technology. Both selecting the cell type and resourcing are the vital parts and members in the cell sheet engineering and its application in which the great potential of the mesenchymal stem cell (MSCs) therapies was reported through the various clinical experiments [1, 2]. Harvesting the cells, after treating the proteolytic enzyme, normally supplies the injectable cell suspensions. It is necessary to mention that the cell surface protein damage causes that the harvested cells demand more time to recover the cell-to-cell and cell-to-extracellular matrix (ECM) interactions, besides the related matrix, adhesion molecules, and receptors [3]. In addition, the prepared 2D cultures of MSCs gradually lose the potential for proliferation, the efficacy of colony-forming, and differentiation ability [4]. Importantly, the success of the traditional cell therapy depends on the regenerative and healing capability, which is reduced due to changing the cell retention and engrafting into the tissue/organs [5]. Considering the mentioned limitations, the researchers started a new way to overcome the aforementioned issues.

The other technology behind cell sheet engineering is designing the responsive polymers as the substrate for cell growth, which includes the advantage of the temperature-responsive properties [6]. Once the polymers are located on the culture surfaces, the cells seeded on top of them are released as the intact and living sheets following the temperature changes without proteolytic enzyme treatment, leading to the high confluence of cells and uniform distribution [7]. In comparison to the conventional scaffold-seeded method, cell sheets technology could preserve native tissue phenotypes and functions, cell-cell conjugations, and secreted ECM [8]. Moreover, the similarity of its composition to the native tissue eliminates the limitations associated with scaffold degradation. The stated approach is applicable for assembling 3D multi-layered structures to mimic complex constructions of the natural tissues [9]. The characteristic features of the cells being used in tissue engineering included potent proliferation, steady passage capacities, ease of collection, and negligible immune rejection responses. In routine cell culture, stem cells are subjected to oxidative stress causing genome, and proteome damage and stimulates senescence and tumorigenesis [10, 11]. The previous studies represented that antioxidant supplementation not only could reduce oxidative stress, but also improve the survival, differentiation, and potential of the stem cells [12–14]. Vitamin C, a water-soluble antioxidant, has a significant effect on collagen expression and ECM biosynthesis [15]. Recent three works from the present study’s lab also appeared the engineering of the different types of sheets including MSCs sheet [23], hepatic sheet [22], and corneal endothelium cell sheet [16]. Ebert et al. concluded that culturing the telomerase-immortalized human mesenchymal stem cells and bone marrow stromal cells (BMSCs) in standard cell culture had impaired antioxidative selenoenzymes. The mentioned enzymes were selenium-depended enzymes that regulated the ROS level. They applied selenite (100 nM vs. 5–10 nM in routine media) to restore the activity of the glutathione peroxidases [GPxs] and thioredoxin reductases [TrxRs] and to decrease ROS accumulation and micronuclei formation [17]. In the current study, the researchers evaluated the feasibility of using the sodium selenite supplementation in the formation of the cell sheet.

Thermosensitive hydrogels are an interesting candidate for biomedical applications since they remain as viscous sols at room temperature but transform into gels upon increasing the temperature to 37 °C [18]. The majority of the thermosensitive polymers represent a lower critical solution temperature (LCST). The most commonly used thermosensitive polymer in cell sheet engineering is poly (N-isopropylacrylamide) (pNIPAAm) with LCST of about 32 °C in aqueous media. It is either hydrophobic or hydrophilic above and below the considered temperature, respectively, and the hydrophilic nature allows the aqueous media to penetrate the interface between the grafted polymer and adherent cell sheet, which facilitates the separation of the cell surface from the culture surface [19]. Researchers have evaluated the different types of copolymers as transformable cell sheet carriers. Among them, the sol-gel transition of triblock copolymer PCL-PEG-PCL (PCEC) has been extensively studied due to its advantages including being FDA-approved, biocompatible, biodegradable, and non-toxic [20–22]. The ratio of the PEG to PCL and their molecular weight have impact on the gelling behavior of the PCEC copolymer. Furthermore, the temperature of the sol-gel transition is directly correlated to the molecular weight of the PEG moiety [23, 24]. According to the obtained knowledge, the present researchers were encouraged to investigate the feasibility of the PCL-PEG-PCL copolymer as a substrate for cell sheet engineering.

Herein, we synthesized the triblock PCEC copolymer, based on the ABA architecture to develop temperature-responsive cell culture dishes. In this regard, we use PEG with a molecular weight of 1500 Da according to the previous studies [25]. In this architecture, PCL and PEG blocks correspond to the A and B blocks of the ABA structure, respectively. Then, the effect of sodium selenite, vitamin C, and Trolox (water-soluble analog of vitamin E) in the osteogenic and adipogenic differentiation, proliferation, and sheet formation of rBMSCs was evaluated.

## Materials and methods

### Synthesis of polycaprolactone-co-poly (ethylene glycol)-co-Polycaprolactone (PCEC) copolymers

The PCEC triblock copolymer was prepared by ring-opening copolymerization of ε-caprolactone (ε-CL, 98%, Aldrich) initiated by polyethylene glycol (PEG, M_n_ = 1500 Da, Aldrich) using stannous octoate (Sn(Oct)_2_, 95%, Aldrich) as a catalyst [26]. Before starting the polymerization reaction, ε-CL monomer was distilled under the reduced pressure at 90 °C after being treated with CaH_2_ for 24 h to remove the inhibitor. Also, PEG was dried in a vacuum oven for 72 h at 70 °C before use, as mentioned previously [27, 28]. The PCEC copolymer was prepared as follow: ε-CL (0.175 mol, 10.0 g) and PEG (0.01 mol, 5.0 g) were added into two necked vessels under a dry nitrogen atmosphere and the reaction system was kept at 120 °C for 1 h. Then, Sn(Oct)_2_ (0.1% Wt of the total mass of monomers) was added to the reaction vessel and continued to stir for about 6 h under the nitrogen flow at 120 °C with a rotating speed of 350 rpm. After the gel formation and production of the light-yellow polymer, a few droplets of HCl 37% were added to terminate the polymerization reaction and the resultant copolymer was cooled to the room temperature. In the end, the obtained PCL-PEG-PCL triblock copolymer was dissolved in dichloromethane and precipitated in the chilled diethyl ether. The yield of the polymerization was obtained at about 93%. The purified polymer was kept in an air-tight bag before use.

### Characterization of PCEC Copolymers

#### Fourier Transform Infrared Spectroscopy (FTIR)

The functional groups of PCEC copolymer were assessed by using the computerized ATR-FTIR spectroscopy (Burker Tensor 27, USA). The spectrum of the sample was recorded from 4000 − 400 cm^− 1^.

#### ^1^H Nuclear Magnetic Resonance analysis (^1^H NMR)

The chemical composition and molecular weight of the copolymer were characterized by ^1^ H-NMR (400 MHz) spectra in CDCl_3_ solvent by using a Spectrospin Avance spectrometer. Chemical shift for the proton of CDCl_3_ is reported at 7.26 ppm.

#### Gel permission chromatography (GPC)

A Shimadzu LC-20 A apparatus along with the refractive index detector was used to determine the weight average molecular weight (Mw) and molecular weight distribution (PDI) of the PCEC triblock copolymers. Tetrahydrofuran (THF) was used as a mobile phase with a flow rate of 1.0 ml/min at 35 °C. The Mw of the copolymer was calculated from the polystyrene standard with narrow molecular weight distribution.

### Investigation of phase transition temperature

The sol-gel-sol phase diagram of the thermosensitive copolymer PCEC in water was studied by using the test tube inverting method in a 4 ml screw-capped vial (diameter 1.1 cm) based on the flow (sol) and nonflow (gel) criterion [29]. The solutions with a given concentration were prepared by rapidly dispersing in 55 °C water followed by cooling in an ice bath. The solutions were kept at 4 °C for 12 h before measurements. To determine the phase transition temperature, the samples were measured from 10 to 60 °C. The samples with no visual flow after inverting the vial for 30 s were considered as a gel.

### Effect of PCEC thickness on cell sheet formation

To assess the effect of PCEC thickness in the cell-sheet formation, two volumes of the polymer gels (0.5- and 1.0 ml) were added into wells of 6-well format with equal diameters. Then, 1.5 × 10^6^ rBMSCs cells were seeded on top of the polymers with different thicknesses and allowed to produce cell sheets for 21 days. The viability and strength of the cells were checked after sheets were released from each well.

### Rat bone marrow-derived mesenchymal stem cells culturing

The experimental procedures were approved by the Animal Ethical Committee of Tabriz University of Medical Sciences with the number of the IR.TBZMED.REC.1399.036. The rBMSCs were isolated according to the previously described protocols [30]. Briefly, about 10^6^ cells of passage 3 were cultured on a T75 tissue culture flask at 37 °C in a humidified atmosphere of 5% CO_2_ containing DMEM supplemented with 10% fetal bovine serum (GIBCO, Invitrogen). The culture medium was changed every 3 days. When cells reached more than 80% confluency, the cells were detached by using 0.25% trypsin / 1 mM EDTA. The cells were aliquoted into three flasks and considered for the next experiments.

### Osteogenic and adipogenic differentiation of rBMSCs

Osteogenic/adipogenic differentiation of rBMSCs was performed according to the previous reports [31]. The rBMSCs were cultured at a density of 5 × 10³cells/cm^2^ on a 6-well plate with a culture medium supplemented with 10% MSC Qualified FBS (GIBCO, Invitrogen), incubating at 37 ^o^C with 5% CO_2_. After 24 h, the cells reached about 60–70% confluency, and then the medium was changed with differentiation media (GIBCO, Invitrogen). Osteogenic differentiation media supplemented with 10% FBS, 50 µM dexamethasone, and 100 nM indomethacin and adipogenic differentiation media contained the 0.05 mM vitamin C, 0.1µM dexamethasone, and 10 mM β-glycerophosphate. Control groups were cultured just with 10% FBS in DMEM. The cultures were incubated at 37 °C in 5% CO_2_ for 21 days and the medium was changed twice a week. At the end of 21st day, the medium was removed and rinsed once with warmed PBS (37 ^o^C). Next, the cells were stained with alizarin red and oil red solution to confirm the osteogenic and adipogenic differentiation, respectively. The images were recorded by using a light microscope.

### MTT assay

First of all, rBMSCs were seeded into 96-well plates at a density of about 10^4^ cells per well and then treated with sodium selenite (Se), vitamin C (Vc), sodium selenite + vitamin C (Se + Vc), and Trolox (Tx, 1, 10, 0.1, 0.01, and 0.001 µM). Their cytotoxic effect on the seeded cells was assessed by MTT mitochondrial metabolism evaluation method [14]. After 1, 3, and 6 days, the treatments were aspirated and replaced with MTT solution (5 mg/mL) followed by incubation for 4 h, then, the medium was removed, and 100 µL DMSO was added to dissolve the formazan crystals; finally, glycine buffer was added. Cell viability was measured at 540 nm by using an ELISA plate reader (Multiskan MK3, Thermo Electron Corporation, USA). Cell growth was compared with the control routine media exposed cells. The results were reported as mean ± SD for three independent samples.

### Fabrication of rBMSCs sheet

First, the 6 well plates were coated by PCEC copolymer, then the seeding of rBMSCs was performed at a density of about 5 × 10^6^ cells per well by applying the routine medium as the control group, and four other groups including the medium supplemented with Vc (50 µg/ml), Se (0.1 µM), Se + Vc (50 µg/ml + 0.1 µM), and Tx (25 µg/ml). The cells were continuously cultured for 2 weeks without passaging, and each medium was replaced every 3 days, cell adhesion, viability, and proliferation were monitored by a phase-contrast microscope (Olympus) until sheets formed and reached confluence. Next, the cell sheets were harvested by temperature reduction by keeping the wells in an ice bath for 5 min. Cell sheets released from the PCEC sol form were observed with an inverted microscope (Olympus).

### Histological analysis of sheets

The harvested cell sheet was rinsed with sterile PBS at 37 ºC, fixed in 4% paraformaldehyde for 1 h at 4 °C in the refrigerator, then, embedded in paraffin and sliced into 5-µm sections. To examine the histology of the prepared cell sheets, hematoxylin and eosin (H&E) staining was performed by common methods, and their photographs were captured by a light microscope (Olympus).

### Field Emission Scanning Electron Microscopy (FESEM)

To assess the morphology and other details of the released sheets, they were processed and visualized by the FESEM method. In this way, they were fixed by a PBS solution of 2.5% glutaraldehyde (pH 7.3) at 4 °C and then 2% osmium tetroxide in the same buffer solution for about 1 h. Subsequently, they were subjected to dehydration procedures in several concentrations of the ethanol and t-butyl alcohol (50, 75, 95, and 100%) for 15 min and then, lyophilized overnight led to the cell sheets by osmium coating. Then, the apical and basal surfaces of the sheets were analyzed by the FESEM.

### Quantitative real-time PCR (q RT-PCR)

The expression level of the stemness genes (Sox2, Oct-4, Nanog), selenium-dependent enzymes (TRX, GPX-1), and aging regulator gene (Sirt1) were measured by the real-time PCR in each released cell sheet. The total RNA was extracted using Trizol reagent (Thermo scientific) according to the manufacturer’s protocols. The cDNA was synthesized by using a Thermo scientific kit (Revert Aid cDNA synthesis kit) and q RT-PCR was done by SYBR green Master Mix (Thermoscientific). The cDNAs, primer, and master mix cocktail were incubated for 3 min at 95 ºC, started loop 40 cycles at 94 ºC for 30 s, 60 ºC for 40 s, 72 ºC for 45 s, closed loop, and finally at 72 ºC for 5 min. The results were reported as fold changes that were calculated by the 2-ΔΔCT method. The sequences of the primers are shown in Table 1.

**Table 1:** The sequences of primers in q RT-PCR.

Gene	Primer sequences (5`→3`)
Nanog	F: GAGACTGCCTCTCCTCCGCCTT R: GTGCACACAACTGGGCCTGA
Oct-4	F: CTGGAGAGGGATGTGGTTCG R: AAGGGACCGAGTAGAGTGTG
Sox2	F: TGGGAGAAAGAAGAGGAGAGA R: CGAAGTGCAATTGGGATGAAA
Sirt-1	F: CGCCTTATCCTCTAGTTCCTGTG R: CGGTCTGTCAGCATCATCTTCC
TRX	F: CTCTTTCCGCACACAGCATA R: CTGTGGGCTCACTGAACAGA
GPX-1	F: GTTCCAGTGCGCAGATACA R: CCAGATACCAGGAATGCCTTAG
GAPDH	F: TCAAGAAGGTGGTGAAGCAG R: AGGTGGAAGAATGGGAGTTG

### Statistical analysis

Statistical analyses were done by using the GraphPad Prism version 8 (GraphPad Software, Inc., La Jolla, CA). All experiments were performed in triplicated and reported as mean ± SD. The two statistical methods, ANOVA or Student’s T-Test were performed for comparing the groups. The significance level was defined as P-values < 0.05.

## Results and discussion

### Synthesis and characterization of PCEC Copolymers

**Fig. 1:**
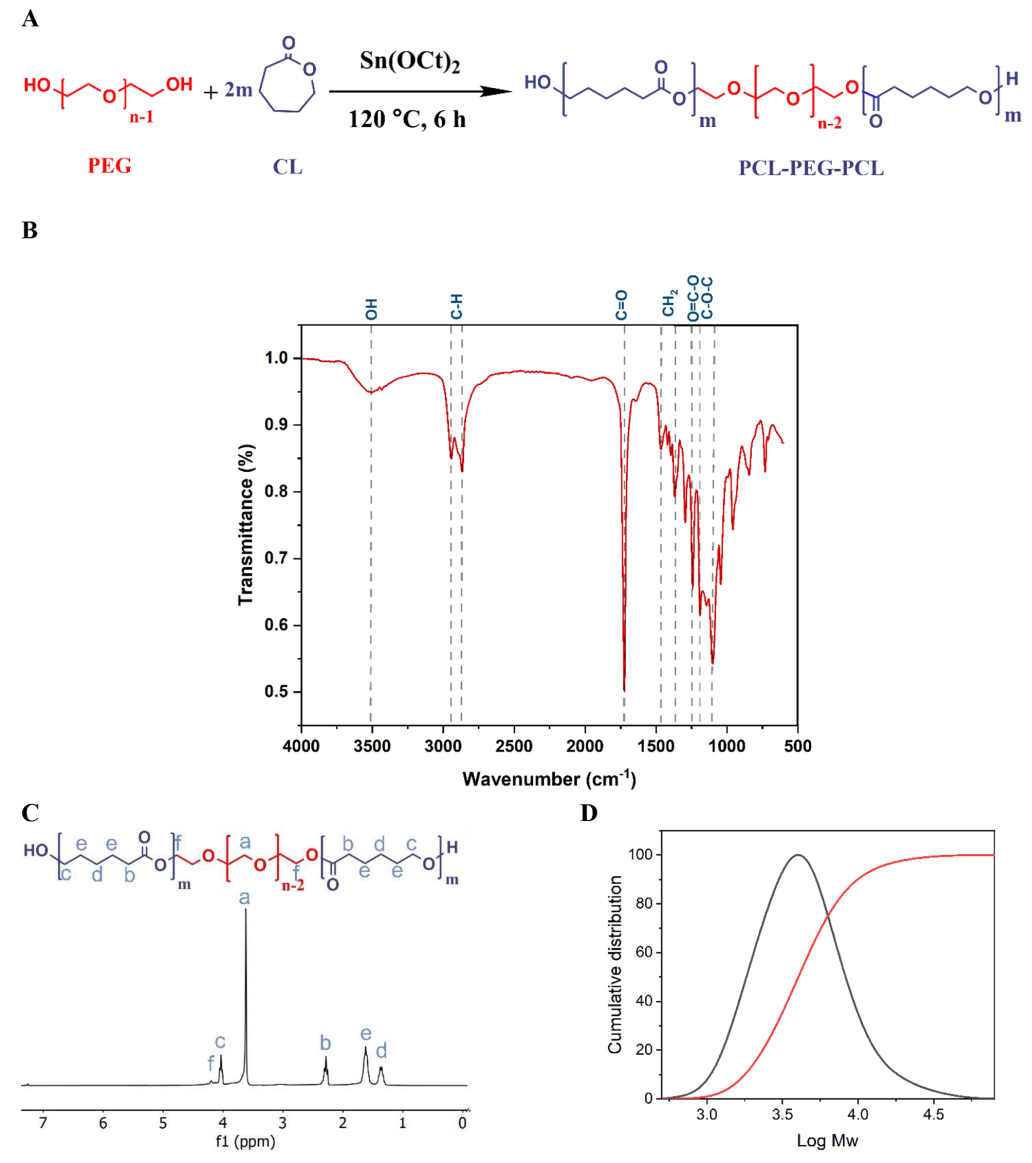
(**A**) Synthesis route for the fabrication of the PCL-PEG-PCL triblock copolymer via the ring-opening copolymerization of ε-caprolactone (ε-CL) initiated by polyethylene glycol (PEG) using stannous octoate (Sn (Oct)_2_) as a catalyst; (**B**) FTIR spectra of the copolymer; (**C**) ^1^H NMR spectra of the copolymer in CDCl_3_; (**D**) GPC profile of copolymer

Through the work, the triblock PCEC copolymer was synthesized and applied as a platform for rBMSCs sleet formation. The synthesis route is depicted in Fig. 1A.

### FTIR Analysis

The FTIR spectrum of the PCEC copolymer is depicted in Fig. 1B. The strong peak at 1724 cm^− 1^ was attributed to the ester carbonyl stretching vibration of the PCL block. Also, the peaks at 1190 and 1101 cm^− 1^ corresponded to the asymmetric and symmetric stretching vibration of the C-O-C in the repeating -OCH_2_CH_2_ units of PEG moiety. The absorption bond around 1242 cm^− 1^ is attributed to the -COO stretching mod [32]. On the other hand, the absorption bonds at 1369 cm^− 1^ and 1465 cm^− 1^ were due to the -CH_2_ bending vibrations [33]. Meanwhile, the aliphatic C-H stretching bond of the PEG and PCL segment was observed at 2868 and 2942 cm^− 1^ [34]. The broad peak around 3499 cm^− 1^ could be assigned as the terminal hydroxyl groups of the copolymer.

### ^1^H NMR Analysis

The chemical structure and ^1^ H NMR spectrum of the PCL-PEG-PCL (PCEC) copolymer is presented in Fig. 1C. The sharp peak at 3.62 ppm is assigned to the repeating CH_**2**_CH_**2**_O- units of the PEG block. The weak peak at 4.20 ppm is attributed to the methylene protons of the -CH_2_C**H**_**2**_OCO- at the end of the PEG linking two PCL blocks. The peaks of the methylene protons in the PCL block was appeared at 1.36 (-COCH_2_CH_2_C**H**_**2**_CH_2_CH_2_O-), 1.62 (-COCH_2_C**H**_**2**_CH_2_C**H**_**2**_CH_2_O-), 2.28 (-COC**H**_**2**_CH_2_CH_2_CH_2_CH_2_O-),4.03 (-COCH_2_CH_2_CH_2_CH_2_C**H**_**2**_O-) ppm [35, 36].

The number average molecular weight (M_n_) of PCL-PEG-PCL copolymers was calculated according to the Eqs. 1 and 2 [36]:

1 $$\frac{{4n - 4}}{{{I_a}}} = \frac{{4m}}{{{I_d}}}$$

2 $$\begin{array}{c}{M_n}\left( {PCL - PEG - PCL} \right) = {M_n}\left( {PEG} \right)\\+ 2{M_n}\left( {PCL} \right) = 44n + 2 \times (114m)\end{array}$$

Where n and m represent the block number of PEG and PCL respectively; I_a_ and I_d_ are the integral intensity of the corresponding peaks at 3.62 and 1.36 ppm, respectively [36]. According to Table 2, with the already known molecular weight of the PEG, M_n_ of the copolymer could be found as well as the PCL/PEG block ratio from the ^1^ H NMR spectrum and compared with the theoretical value calculated from the feed ratio. The M_n_ and PCL/PEG block ratio was 4236 and 1.82:1, respectively.

**Table 2:** Characterization of the PCEC Copolymer Synthesized in the Present Work

Copolymer	Yield (%)	PCL/PEG ^a^ (Theoretical)	M_n_^a^ (g/mol) (Theoretical)	PCL/PEG ^b^ (Calculated)	M_n_ (g/mol) (^1^ H NMR)	M_w_^c^ (g/mol)	PDI ^d^
PCL-PEG_**1500**_-PCL	93	2:1	4500	1.82:1	4236	5417	1.279

^a^ Theoretical value, calculated according to the feed ratio; ^b^ Calculated from ^1^ H NMR results based on the PEG block (4 H, 3.62) and PCL block (4 H, 2.28) of the copolymer; ^c^ Weight average molecular weight (M_w_) calculated from GPC; ^d^ PDI of the block copolymer calculated from M_w_/M_n_.

### GPC Analysis

The molecular weight and molecular weight distribution of the PCEC triblock copolymers were also determined by GPC. Figure 1D represented the GPC chromatogram of the synthesized copolymers. The polymer exhibited the unimodal pattern with a polydispersity index of 1.279 in GPC analysis. The elute time has a negative correlation with the length of the polymer chains. So, compared with the GPC curve of polystyrene, the significant reduction in the elution time indicated a remarkable increase in the molecular weight after polymerization [36]. Table 2 shows the different molecular weights of the block copolymer. The distinction between the theoretical and measured M_n_ of the copolymer can be attributed to the polydisperse nature of the synthesized copolymer, which leads to the relatively broad symmetric distribution as a result of producing the different lengths of the polymer chains. Therefore, the measured number average molecular weight of the block copolymer reduces in comparison with the theoretical value [37].

### Sol-gel transition temperature

**Fig. 2:**
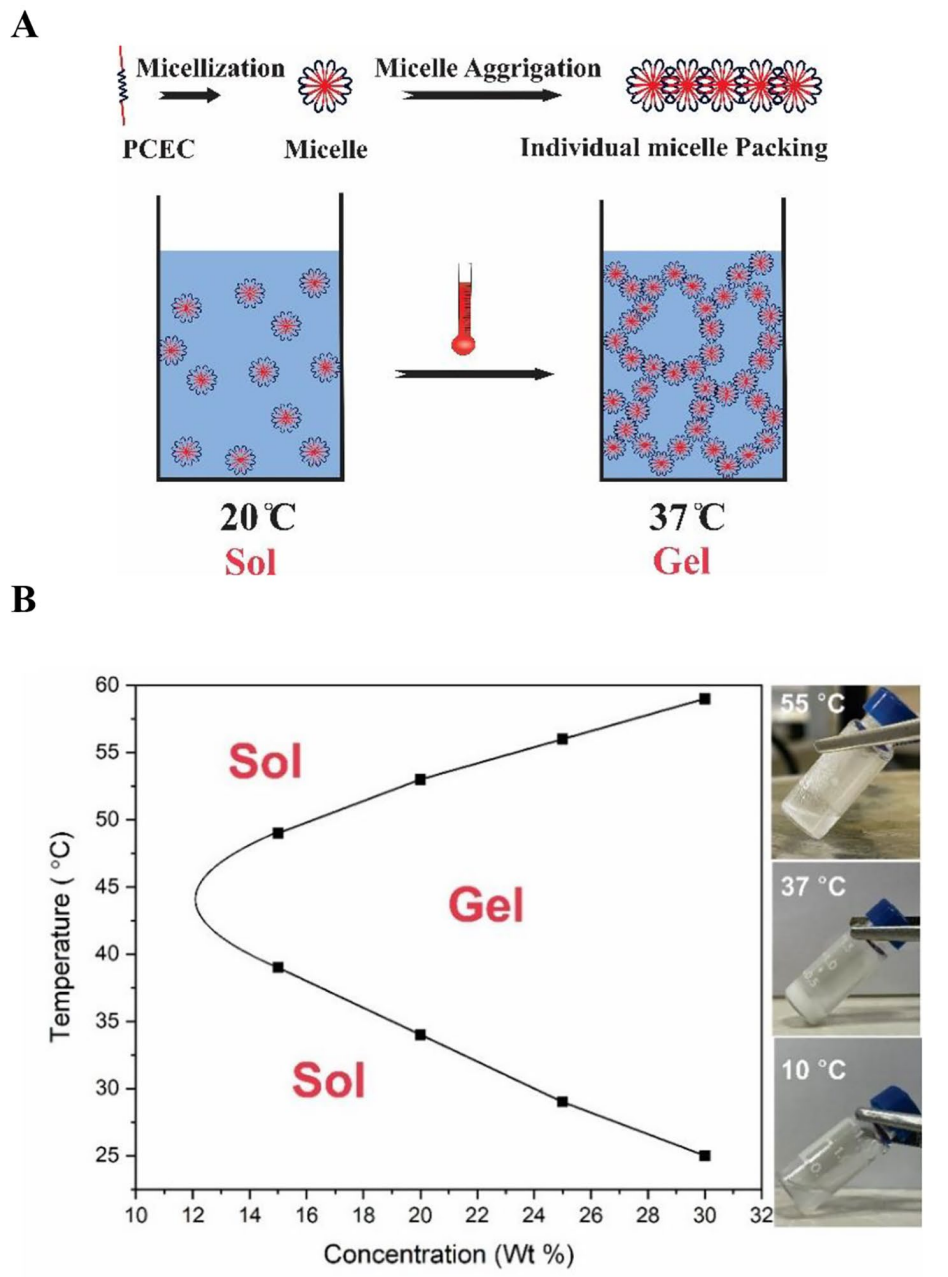
(**A**) The schematic representation of thermos-responsive PCEC hydrogels preparation; (**B**) Sol-gel-sol phase transition diagram and photograph of PCEC hydrogels (20%wt) at 10 °C, 37 °C, and 55 °C showing thermal sensitivity

As it is shown in Fig. 2A, thermo-gelation mechanism of PCEC copolymer has been attributed to micelle aggregation, which is a microscopic phenomenon as a result of a thermos-responsive self-assembly behavior. Micellization is a unique property of an amphiphilic polymer in an aqueous solution. So, when the temperature of the PCEC solution reached the Critical Micelle Temperature (CMT), the polymer chains were converted to the micelle form. In that situation, named sol state, micelles are small in size and freely flowing in the aqueous solution. For conversion from micelles to hydrogels, the minimum concentration and temperature are required to be denoted as Critical Gelation Concentration (CGC) and Critical Gelation Temperature (CGT), respectively. The micelles begin to be packed together tightly and form a 3D network as soon as reaching the above conditions. Conceding that, the 3D network entraps water inside the pores, and sol to gel transition can be observed. Once the temperature increases, the hydrophobic force derives micelle aggregation through the individual micelle packing mechanism [38, 39].

The PCEC copolymer represented the temperature-dependent sol-gel-sol transition and concentration-dependent critical gelation concentration (CGC) that can be depicted in Fig. 2B. The concentrations for the present study were chosen in a way that they were higher than the Critical Micelle Concentration (CMC) of PCEC, which is 0.03 mg/ml [32]. Based on the lower sol-to-gel-transition temperature, the proper concentration for the fabrication of the cell sheet was 20% wt. At 37 °C, the polymer was in the gel form, which was a suitable platform for cell proliferation, adhesion, and spreading. Once the cell sheet is fabricated, it will be detached from the polymer surface by lowering the temperature and transitioning to the sol form without the need for the proteolytic enzymes.

### Culture of rBMSCs

**Fig. 3:**
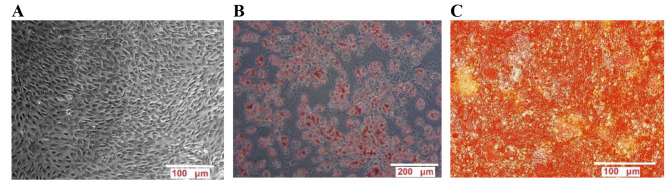
(**A**) rBMSCs as spindle-shaped cells in passage 3. (**B**) Lipid droplets as red color (oil red staining). (**C**) Extracellular calcium deposits as orange color (Alizarin red staining)

The isolation and characterization of the rBMSCs used in the current study were described through the previous research done by the present researchers [30]. The multipotency of the rBMSCs as a key identity marker in stem cells was confirmed via the detection of the osteogenesis and adipogenesis potential. Both the osteogenic potential and adipogenesis of the rBMSCs were evaluated after differentiation induction. In that way, the alizarin red and oil red staining represented the deposition of the minerals and oil droplets around the differentiated cells (Fig. 3).

### Study of cell proliferation by MTT assay

**Fig. 4:**
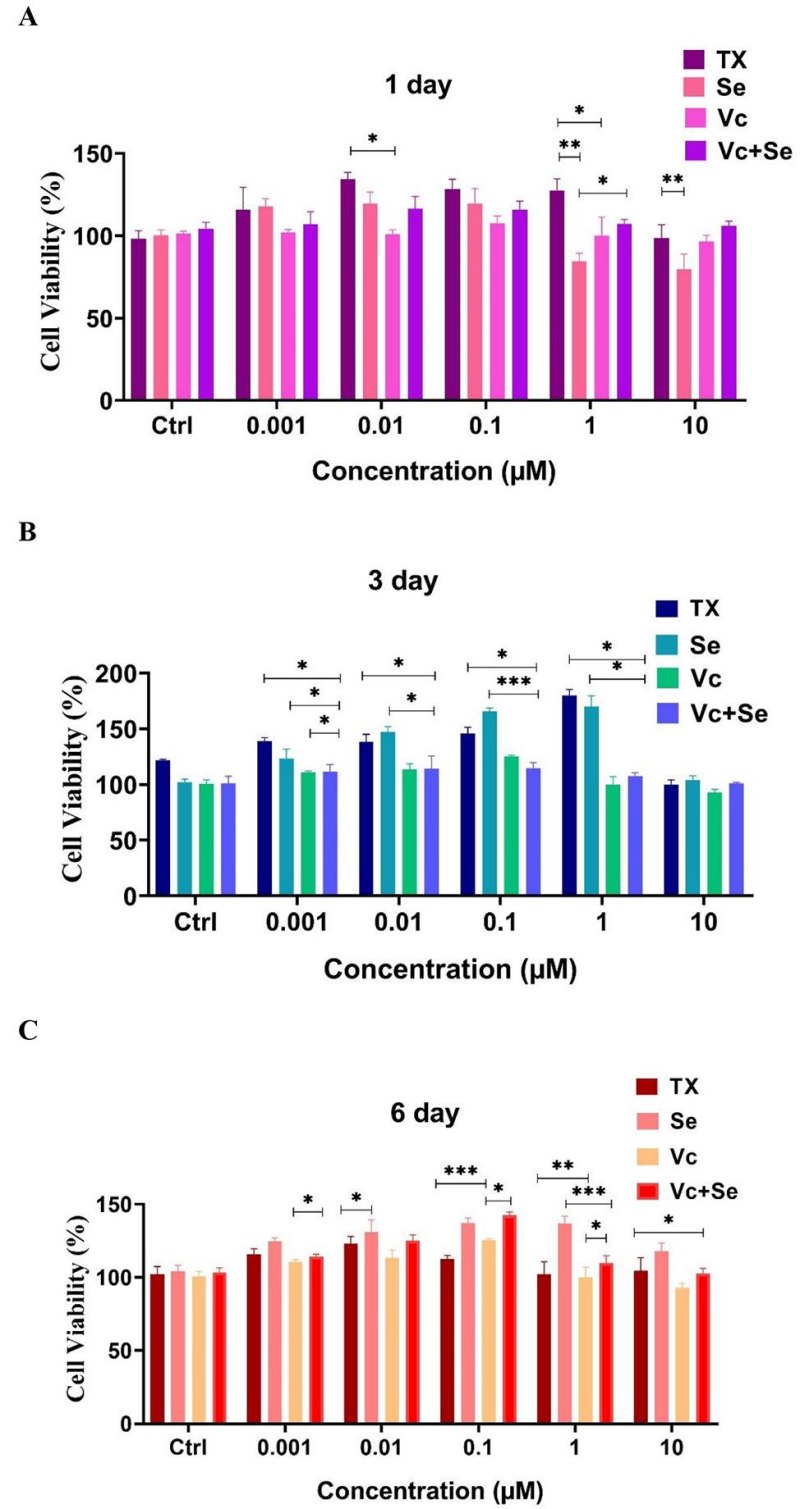
Proliferation evaluation of rBMSCs treated with different concentrations of Se, Se + Vc, and Tx on (**A**) day 1, (**B**) day 3, and (**C**) day 6. The error bars represent the mean ± SEM of three independent experiments. *P < 0.05, **P < 0.01, ***P < 0.001

The survival rate and proliferation of the rBMSCs treated with different concentrations of antioxidants ranging from 0.001 to 10 µM were evaluated by MTT assay. The results showed that Se, Vc, Se + Vc, and Tx (as a reference antioxidant) could induce cell growth and proliferation at any concentration after 1 and 3 days of the treatments. While, by increasing the duration of the treatment to 6 days, the rate of proliferation decreased. Also, Se as an alternative for Vc (a common antioxidant) induced proliferation even better than Vc by increasing incubation time to 6 days. The co-treatment of the rBMSCs with both Se and Vc presented a synergistic effect on cell proliferation only at day 1, but, as the incubation time increases, Se alone performs better than co-treatment with Se and Vc. The change in cell proliferation at different concentrations of Se, Vc, and Tx and time can be seen in more detail in Fig. 4. In the present study, 0.1 µM of selenium was chosen for sheet production which is the optimal concentration for MSCs according to the Ebert et al. study [17].

### Cell sheet formation method

**Fig. 5:**
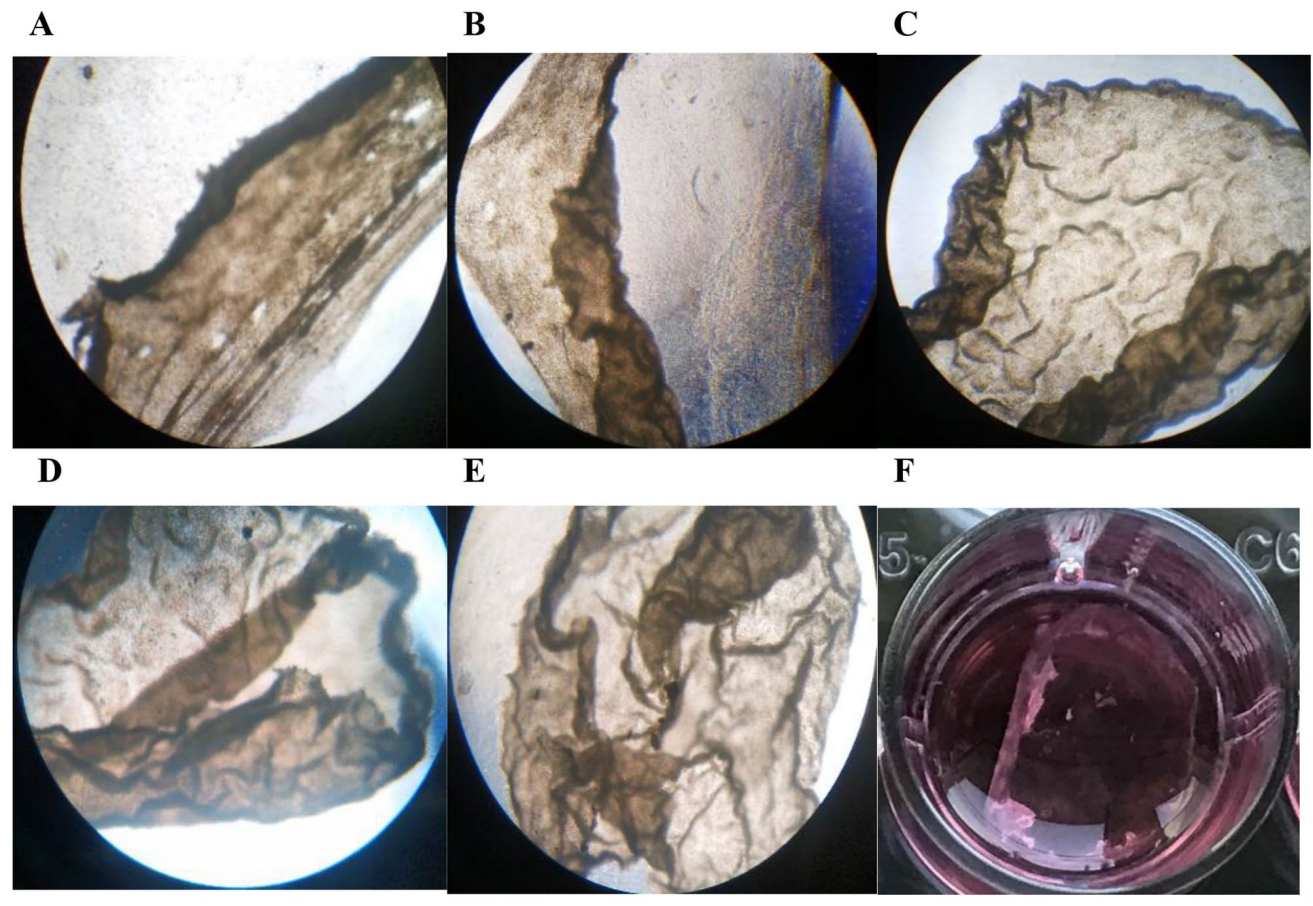
Microscopic appearance of the obtained cell sheets using additional components. (**A**) routine medium. (**B**) medium enriched with Vc. (**C**) medium enriched with Se. (**D**) medium containing Se + Vc. (**E**) medium containing Tx. (**F**) image of released cell sheet from sodium Se. Magnification: 4X

To optimize sheet formation, Vc, or Se, was added to the media. The role of Vc as the main factor for producing robust sheets has been approved previously [14]. The impacts of 0.1 µM Se in the production of cell sheets was evaluated through the current investigation. The rBMSCs could form sheets in the osteogenic differentiation medium, while no sheets were formed in the DMEM medium alone. Figure 5 shows the sheets formed in five induction media. It was found that the sheets obtained in the presence of Se were robust compared with other experimental groups in which the thin layers of the cell sheet were released after 19 days of induction.

### Characterization of cell sheets

**Fig. 6:**
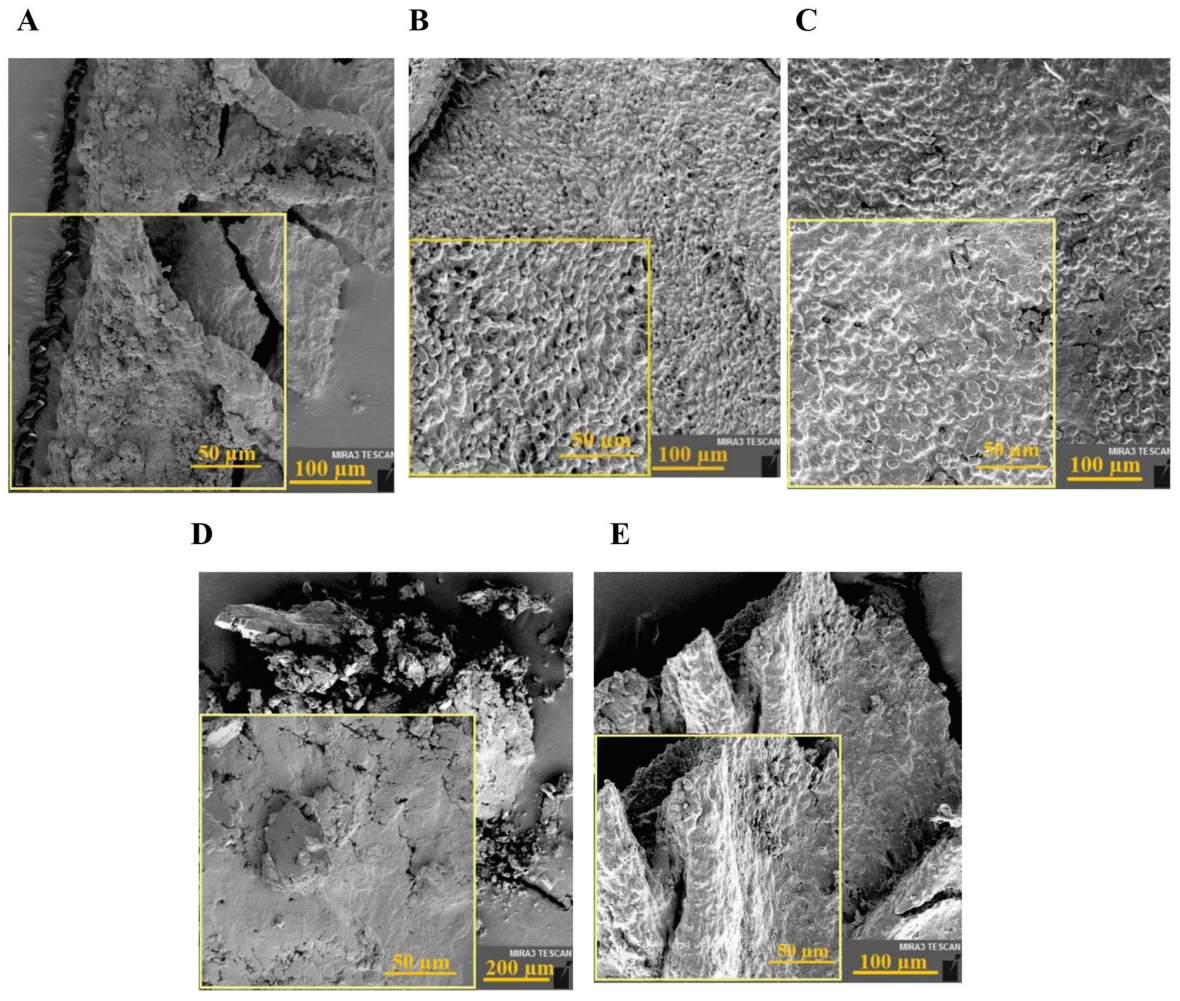
FESEM images of the cell sheets to assess the impact of (**A**) Control media that showed the fragile cells; (**B**) Vc that produced tissue-like sheets; (**C**) Se that produced tissue-like sheets; (**D**) Se + Vc that showed the fragile sheets; (**E**) Tx that produced tissue-like sheets

**Fig. 7:**
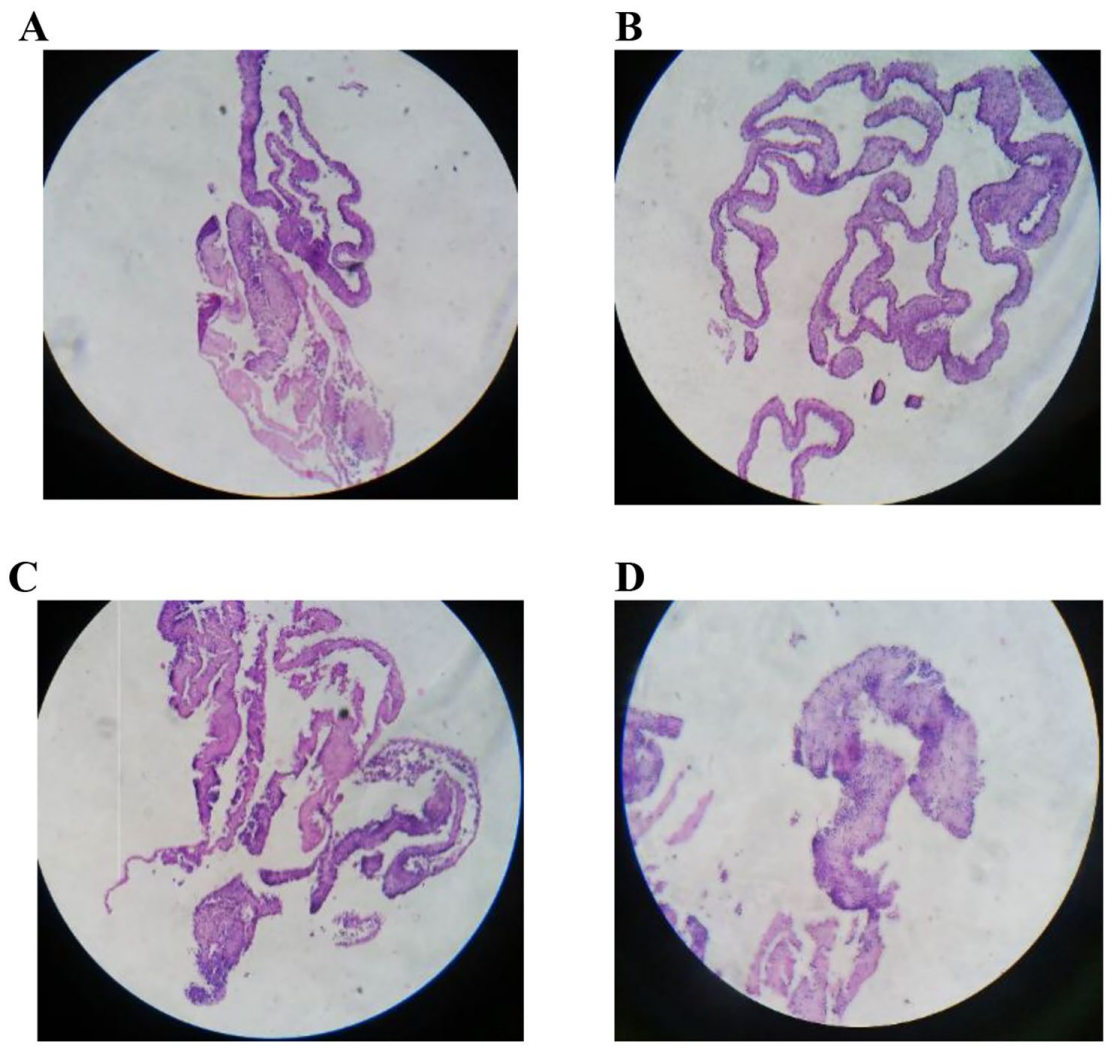
Cross-sectional H&E staining of the cell sheets, in which several layers of the cells and a thick tissue-like structure could be created by stacking these layers. (**A**) H&E staining of the sheet prepared in the presence of Se; (**B**) H&E staining of the sheet prepared in the presence of Vc; (**C**) H&E staining of the sheet prepared in the presence of Se + Vc; (**D**) H&E staining of the sheet prepared in the presence of Tx (Magnification: 10X)

Cell sheets were released after being placed on an ice bath due to the polymer transition from gel to sol. The FESEM images in Fig. 6 represents tissue-like and intact sheets as well as the distinguished cells. Figure 7 represents the cross-sectional H&E staining of the fabricated cell sheet. In these images, the nucleus were illustrated as purple (due to the interaction of hematoxylin with phosphate groups) and the cytoplasm was as pink color (due to the binding of positive eosin with lysin or arginine residues).

### Senescence-Associated Beta-Galactosidase activity

**Fig. 8:**
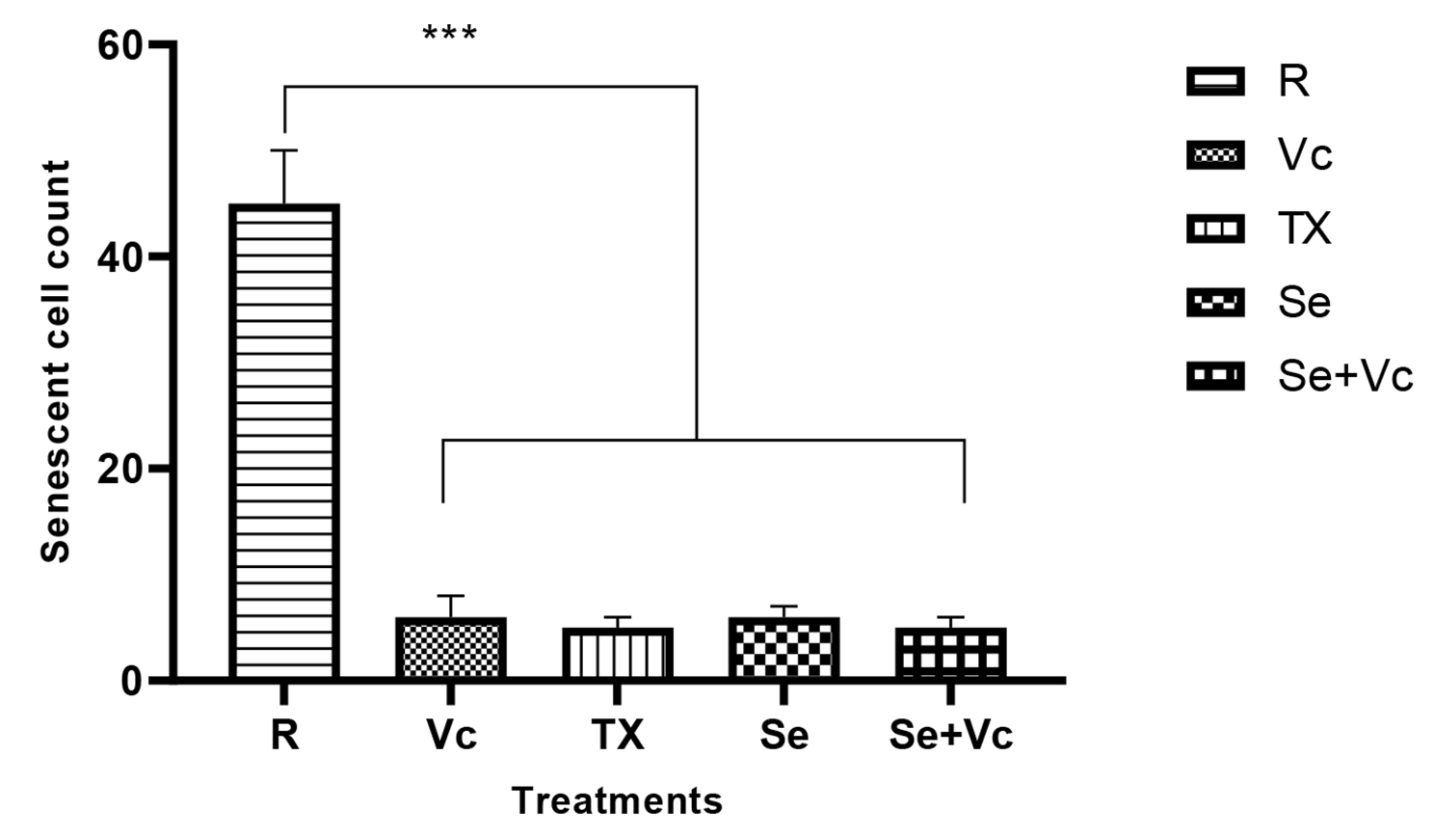
Effects of Vc, Se, and Tx on the cell senescence of the rBMSCs. The addition of Se and Vc to culture media could reduce the senescence compared to the control cells (passage 6), (R: Routine medium)

In general, the activity of galactosidase-beta is determined by staining the cells with its substrate 5-bromo-4-chloro-3-indolyl-beta-d-galactopyranoside (X-gal). The nonsenescent cells were not stained as the lysosomal activity was suppressed. The application of complete medium induced the senescence-associated beta-galactosidase, while treating by Vc and Se reduced the senescent cells. The frequencies of senescent cells which showed beta galactosidase positive (blue/green spots) upon facing X-gal were counted in four main microscopic directions. Then mean ± SD of each treatment-positive cells were plotted (Fig. 8).

### Q RT-PCR analysis

**Fig. 9:**
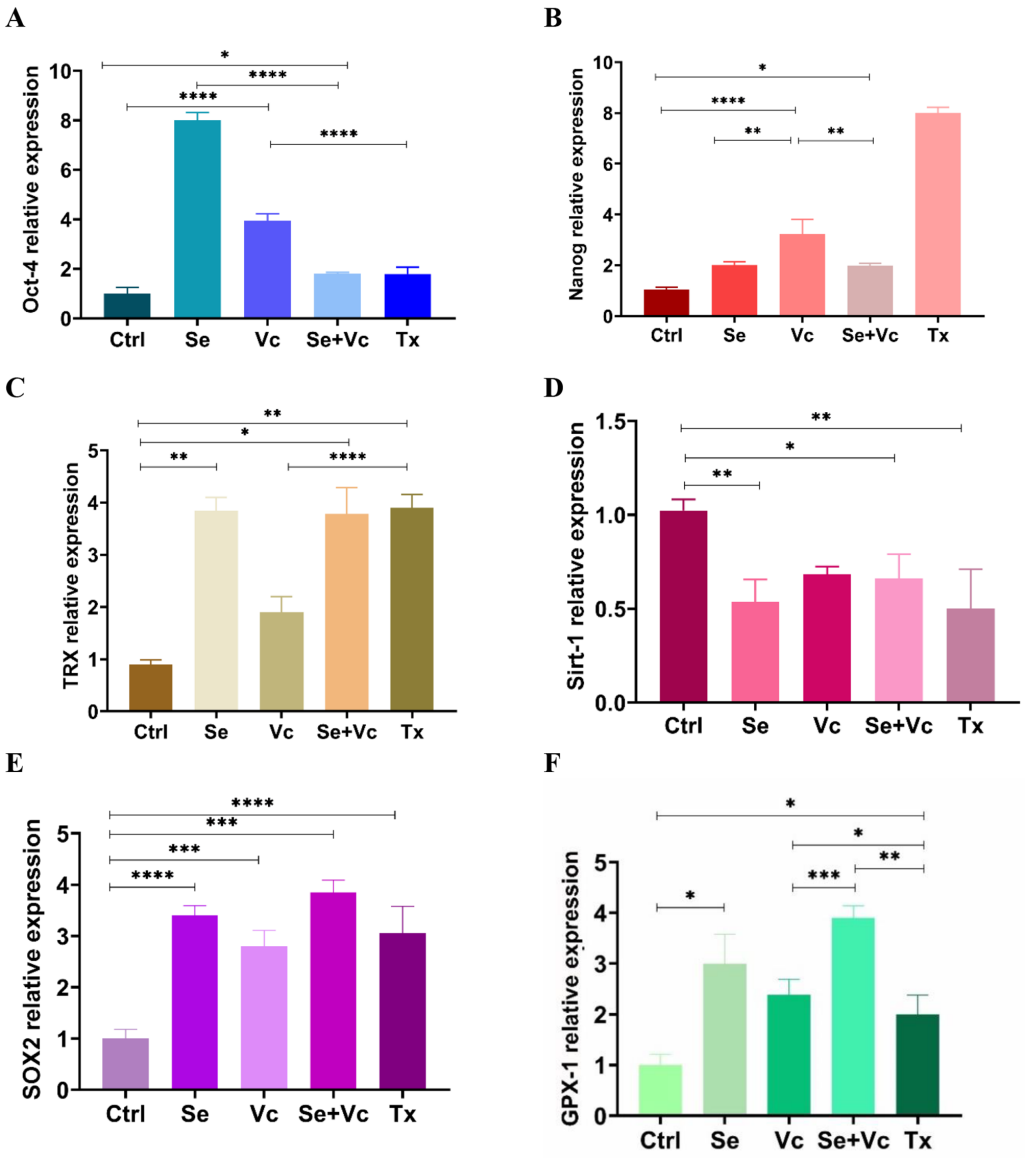
The expression levels of the desired genes. Relative expression of (**A**) Oct-4, (**B**) Nanog, (**C**) TRX, (**D**) Sirt-1, (**E**) SOX2, and (**F**) GPX-1 in the released cell sheets by real-time PCR analysis; the high level of the expression in Oct-4, Nanog, TRX, SOX2, and GPX-1 and low level of the expression in Sirt-1 were found when treated with Vc and Se compared to the control media. The values were normalized to GAPDH. (*p < 0.05, **p < 0.01, and ***p < 0.001)

The results of q RT-PCR analysis showed that the expression level of the stemness-associated genes, Oct-4, Sox2, and Nanog increased when treated with Se and Vc compare to the control group and in this regard, the results of Se were comparable and even better than Vc. In addition, the expression level of Sirt-1, as a senescent marker, is reduced in the presence of Se and Vc, and in this case, Se performed better than Vc. Furthermore, Se and Vc caused the upregulation of TRX, a selenium-containing enzyme, which reduces oxidative stress and intracellular ROS and GPX-1. The change in the expression level of the desired genes can be seen in more detail in Fig. 9.

## Discussion

In this study, a biocompatible triblock PCEC copolymer was synthesized and used as a coating substrate for the fabrication of the rBMSCs cell sheet. Also, the effects of antioxidants including vitamin C, sodium selenite, and Trolox in the production of the rBMSCs sheets were evaluated. Among the chemically synthesized polymers, the most commonly used one is poly (N-isopropylacrylamide) (pNIPAAm) [14] which could be modified by using its monomers or tunning their concentrations. The present article’s aime is to study another alternative of pNIPAAm as a substrate for the fabrication of the rBMSCs sheet. The possibility of using PCEC copolymer was evaluated as an innovative substrate for the cell sheet engineering because it is not only cost-effective but also FDA-approved [40]. Some amphiphilic PEG/PCL copolymers with suitable molecular weights can dissolve in water to form free-flowing sols at low or ambient temperatures, and at the physiological temperature of 37 °C, spontaneously form hydrogels called thermogels which contains an internal structure of a micelle network. It can be concluded that the thermal-induced gelation comes from a hydrophilic − hydrophobic balance of the amphiphilic copolymer. On the bases of the above background and the result of a previous study, the PEG was chosen in the present study with molecular weight 1500 Da in the synthesis of PCEC copolymer to see the phase transition at physiological temperature. To do so, various concentrations of the aqueous polymer solution were investigated by using the test tube inverting method. The formation of the hydrogel was observed at 37 °C in 20% wt aqueous polymer solution as it can be shown in Fig. 2B.

According to the previous studies [14, 41–44], the supplementation of Vc to the culture media induces more proliferation and collagen/ECM deposition, sheet development, and harvest. The results of the Erbert et al. study [17] showed the cytoprotective and antioxidant properties of the sodium selenite on rBMSCs. Also, Park et al. [45], reported that selenium addition to the medium could improve the proliferation and potency of 3T3 cells. Another work documented the positive effect of sodium selenite on collagen synthesis [46]. Through the current study, the sodium selenite was supplemented in the medium to encourage the rBMSCs to proliferate and secrete large amounts of collagen, which leads to the rapid formation of the cell sheet. Furthermore, it was compared with the effects of other supplements like Vc, Vc + Se, and Tx on cell sheet formation. On top of that, the histological and biological characteristics of the prepared cell sheets were studied through H&E staining, FESEM, real-time PCR analysis of stemness genes and selenium-related enzymes transcription, and beta-galactosidase senescence detection. The results for cell sheets preparation in the presence of Tx, Vc, Se, and its combination with Vc showed that the sheets formed in the presence of Se were comparable or even better than the sheets formed in the presence of Vc. Therefore, for the preparation of rat bone marrow mesenchymal stem cell sheets it was realized through the present study that the Se supplementation could be suitable for future applications.

Among the 25 proteins of selenoproteome [47], Cytosolic glutathione Peroxidase (GPx-1) and Thioredoxin reductases (TRX) are the most well-known selenoproteins, which act by reducing the glutathione or thioredoxin, respectively, to eliminate peroxides toxicity. In addition, it can influence the flow of signaling pathways in cells in favor of cytoprotection [48].

In this research, selenium treatment increased the expression of selenoproteins including GPX-1 and TRX which is consistent with recent studies. They showed that the activity of the mentioned enzymes rose after being treated by selenium, as cytoprotective against oxidative stress and supporting DNA repair [49, 50].

As it has been understood, it has been the first report of the use of Se to induce the rBMSCs to construct intact sheets. The proliferation results of the rBMScs on days 1, 3, and 6 showed that Se could induce proliferation better than Vc. Another fundamental aspect of the addition of these antioxidants improved the quality of cell sheets by reducing senescence (reduction of Sirt-1 expression). Application of both Vc and Se can increase the expression of the stemness genes (such as Nanog, Sox2, and Oct4); however, Se can work better than Vc alone. These results could address the application of these engineered sheets in cell therapy. Selenite supplementation of the rBMSC cultures seems to be an important countermeasure to restore their antioxidant capacity and reduce the cellular damage in tissue engineering and transplantation procedures.

In general, the obtained data from the preset study could confirm that Se can be used to prevent the senescence of MSCs, increase their antioxidant potential and proliferation and induce sheet formation. Additionally, the biocompatibility of PCEC along with having an appropriate sol-to-gel temperature candidate the mentioned polymer to be utilized as a thermosensitive coating in terms of the cell sheet technology.

## Conclusion

The use of smart hydrogels based on the external stimuli could hold the great promise for application in the field of cell sheet technology. Therefore, the biodegradable and FDA-approved PCEC copolymer was synthesized and used as a thermos-responsive scaffold for the fabrication and release of the rBMSCs sheets based on the sol-gel phase transition temperature. The researchers through the present study selected the PEG with the molecular weight 1500 Da in the synthesis of PCEC copolymer to see the phase transition at the physiological temperature. The rBMSCs were cultured and treated with several antioxidant supplementations in media (including Vc, Se, and Tx) to produce a suitable cell sheet. The results of the MTT assay appeared that Se, as an alternative for Vc (a common antioxidant), induced proliferation even better than Vc by increasing the incubation time to 6 days. Also, Se and Vc represented synergistic effects on the 1st day but by increasing the duration of the treatment, Se induced cell growth better than Vc. The addition of the Se and Vc to culture media could reduce the senescence compared to the control cells. Like Vc, Se up-regulated Oct-4, Nanog, TRX, SOX2, GPX-1, and down-regulated Sirt-1 compared to the control media. The most important role of Se is to improve cell sheet formation; and in the present study, it was found that the use of 0.1 µM of Se could accelerate sheet formation compared to Vc. It is suggested that these sheets need further in vivo analysis and clinical studies to approve their effectiveness as the scaffold-free and safe sheets.

## Data Availability

All data generated or analyzed during this study are included in this published article.
